# Lobar Distribution of COVID-19 Pneumonia Based on Chest Computed Tomography Findings; A Retrospective Study

**Published:** 2020-04-18

**Authors:** Sara Haseli, Nastaran Khalili, Mehrdad Bakhshayeshkaram, Morteza Sanei Taheri, Yashar Moharramzad

**Affiliations:** 1 Chronic Respiratory Diseases Research Center, National Research Institute of Tuberculosis and Lung Diseases (NRITLD), Shahid Beheshti University of Medical Sciences, Tehran, Iran.; 2 School of Medicine, Tehran University of Medical Sciences, Tehran, Iran.; 3 Iranian Society of Radiology, Tehran, Iran.; 4 Department of Radiology, Shohada-E-Tajrish Hospital, Shahid Beheshti University of Medical Sciences, Tehran, Iran.

**Keywords:** COVID-19, pneumonia, viral, tomography, X-ray computed, lung injury

## Abstract

**Introduction:**

Computed tomography (CT) imaging has quickly found its place as a beneficial tool in the detection of coronavirus disease 2019 (COVID-19). To date, only a few studies have reported the distribution of lung lesions by segment. This study aimed to evaluate the lobar and segmental distribution of COVID-19 pneumonia based on patients’ chest CT scan.

**Methods:**

This was a retrospective study performed on 63 Iranian adult patients with a final diagnosis of COVID-19. All patients had undergone chest CT scan on admission. Demographic data and imaging profile, including segmental distribution, were evaluated. Moreover, a scoring scale was designed to assess the severity of ground-glass opacification (GGO). The relationship of GGO score with age, sex, and symptoms at presentation was investigated.

**Results:**

Among included patients, mean age of patients was 54.2 ±14.9 (range: 26 - 81) years old and 60.3% were male. Overall, the right lower lobe (87.3%) and the left lower lobe (85.7%) were more frequently involved. Specifically, predominant involvement was seen in the posterior segment of the left lower lobe (82.5%). The most common findings were peripheral GGO and consolidation, which were observed in 92.1% and 42.9% of patients, respectively. According to the self-designed GGO scoring scale, about half of the patients presented with mild GGO on admission. GGO score was found to be equally distributed among different sex and age categories; however, the presence of dyspnea on admission was significantly associated with a higher GGO score (p= 0.022). Cavitation, reticulation, calcification, bronchiectasis, tree-in-bud appearance and nodules were not identified in any of the cases.

**Conclusion:**

COVID-19 mainly affects the lower lobes of the lungs. GGO and consolidation in the lung periphery is the imaging hallmark in patients with COVID-19 infection. Absence of bronchiectasis, solitary nodules, cavitation, calcifications, tree-in-bud appearance, and reversed halo-sign indicates that these features are not common findings, at least in the earlier stages.

## Introduction

An outbreak of pneumonia associated with a novel coronavirus, now commonly known as SARS-CoV-2, emerged in Wuhan, China in December 2019. Patients infected with this novel virus manifested with symptoms of severe pneumonia, including fever, fatigue, dry cough, and acute respiratory distress (1). Since the announcement of COVID-19 by the World Health Organization (WHO) as a pandemic, elimination of this disease has turned into a global health priority (2). However, without optimizing our knowledge about the efficient diagnosis and treatment of this disease, increase in infection and mortality rates is inevitable.

Primary investigations showed that reverse transcriptase- polymerase chain reaction (RT-PCR) is associated with a low detection rate at initial presentation, as well as being associated with several weaknesses such as limited number of kits, possibility of false negative RT-PCR results, and an undesirable delay in diagnosis (3). Thus, it was essential to find a complementary strategy for time-efficient detection of this disease. Imaging quickly found its place as a critical tool, aiding in the diagnosis of patients suspicious for COVID-19 infection. So far, the literature has mainly emphasized on the role of chest computed tomography (CT) scan for screening and identifying COVID-19 patients, as well as evaluating severity and disease progression. In comparison to radiography, chest CT scan imaging is a more reliable and sensitive method to diagnose and assess COVID-19, especially in epidemic regions (4). Evaluating chest CT findings in combination with clinical symptoms and laboratory tests aids in the medical decision making for COVID-19 disease. In many regions of Iran, CT scan is more available than RT-PCR, the current standard confirmatory test of COVID-19. In these regions, chest CT is used as one of the first modalities for initial evaluation of suspected cases, aiding in prompt management before confirmation of diagnosis by PCR (5, 6). 

The primary findings of COVID-19 on CT imaging are those of atypical pneumonia or organizing pneumonia with significant overlap with other acute lung injuries such as viral infection (especially influenza), drug toxicity, vasculitis, and connective tissue disease (7). 

In a recent study evaluating prediction models for diagnosis and prognosis of COVID-19 infection, one of the mentioned limitations was that reported data from outside of China is scarce (8). Moreover, although multiple studies have been published reporting CT-imaging features of COVID-19 pneumonia; few studies have investigated the distribution of lung lesions by segment. In this study, we aim to evaluate the pattern of COVID-19 pneumonia segmental involvement, by presenting chest CT-scan findings of 63 patients. Also, we have designed a novel scoring scale to report the extension and severity of ground-glass opacification (GGO) in our patients and assess its association with age, sex, and presenting symptoms. 

## Methods

***Study design and participants***

This was a retrospective study performed on 63 adult patients with a final diagnosis of COVID-19 who had undergone chest CT-scan in a tertiary referral hospital in Tehran, Iran, from February to March, 2020. All patients had presented within one week of symptom onset. Diagnosis of COVID-19 was confirmed by a positive RT-PCR for SARS-CoV-2 obtained from a nasopharyngeal swab specimen. The study protocol was approved by the Ethics Committee of Shahid Beheshti University of Medical Sciences (code: IR.SBMU.NRITLD.REC.1399.028). Due to the retrospective nature of the study and no potential risk of harm to patients, the institutional review board (IRB) of Shahid Beheshti University of Medical Sciences waived written informed consent. Also, patients’ personal information was de-identified and kept confidential.

***Participants***

Patients were consecutively selected. The exclusion criteria were age < 18 years, asymptomatic cases with only abnormal imaging, and history of previous underlying lung disease. 

***Data gathering***

Demographic data including patients’ age, sex, and initial symptom at presentation was collected. All patients had undergone at least one non-contrast chest CT-scan with reconstructions of the volume at 1 mm to 2.5 mm slice thickness on the first day of admission before receiving any antiviral treatment. In patients who had more than one chest CT image, only the initial CT scan (on admission) was evaluated in this study. CT images were reviewed by three board certified radiologists using a viewing console. All of the radiologists were blinded to the lab data, clinical features, and patients’ diagnosis. First, imaging findings were interpreted independently and then, final decision was made by consensus. No negative control cases were included in this study.

Imaging features including type of opacity, GGO severity score, and presence of other abnormal findings (such as pleural effusion, interlobular septal thickening, cavitation, reticulation, calcification, bronchiectasis, presence of discrete nodules, and reversed halo sign) were assessed. GGO was defined as increased lung attenuation with preservation of bronchial structures and pulmonary vessels, and consolidation was defined as opacification with obscuration of vascular margins and airway walls. Reversed halo sign was considered a focal rounded GGO surrounded by a ring-like consolidation.

***GGO scoring***

 The extension of GGO was measured based on the following scoring scale: Involvement of less than 1/3 of segments=1; Involvement of 1/3 to 2/3 of segments=2; Involvement of more than 2/3 of segments=3. The greatest score was considered as the final score. 

***Statistical Analysis***

Categorical variables are reported as number (percentages) and continuous variables are reported as mean ± standard deviation (SD). We evaluated the association of segmental distribution with the age group and sex of patients by conducting Chi-square or Fisher’s exact test. Mann-Whitney U test or Kruskal-Wallis test was used to assess the relationship of GGO score with age group, sex, and common initial symptoms (fever, dyspnea, and cough) at presentation. All statistical tests were performed using SPSS v.23 (IBM Inc., Chicago, IL, USA). Statistical significance was fixed at 0.05. 

## Results

***Baseline characteristics of participants***

A total of 63 patients (38 male and 25 female) were included. Mean age of patients was 54.2 ±14.9 (range: 26-81) years old (60.3% male). The most common symptoms at presentation were fever (71.4%), dyspnea (52.3%), and cough (47.6%), respectively. Table 1 shows patients’ baseline characteristics. 

***Chest CT findings***

All patients had abnormal chest CT scan consistent with viral pneumonia. Table 2 shows the frequency of lung lesions based on segmental involvement and also the frequency of different imaging features. The most common imaging feature among our patients was peripheral GGO (92.1%) and consolidation (42.9%). 50.7% of the patients had a mild GGO. Pleural effusion was seen in only one patient. Tree-in-bud appearance, cavitation, reversed halo sign, reticulation, calcification, bronchiectasis, and nodules were not identified in any of the cases. 

Overall, lower lobes of both sides were found to be more frequently involved in our patients. Predominant lobar involvement was seen in the posterior segment of the left lower lobe (82.5%) followed by the superior segment of the right lower lobe (77.8%) and the lateral segment of the left lower lobe (76.2%), respectively. On the other hand, medial segment involvement of the right middle lobe was the least reported finding (17.5%). 

***Segmental involvement based on age and sex***

The inferior lingula (p = 0.012) and the apicoposterior segment of the left lower lobe (p = 0.045) showed a significantly higher involvement among females. Moreover, the anterior (p = 0.017) and posterior (p = 0.043) segment of the right upper lobe, the lateral (p = 0.019) and medial (p = 0.015) segment of the right lower lobe, and the superior segment of the left lower lobe (p = 0.029) were more commonly involved in patients ≥60 years old. 

***GGO score***

GGO score was found to be equally distributed among sex (p = 0.098) and age (p = 0.846) categories. Existence of dyspnea at presentation was significantly associated with a higher GGO score (p= 0.022) 

## Discussion

In this study we have reported chest CT imaging results of patients with laboratory-confirmed COVID-19 pneumonia. Our results revealed that the most common finding was peripheral GGO followed by consolidation. Cavitation, reticulation, calcification, bronchiectasis, tree-in-bud appearance, reversed halo-sign, nodules and pleural effusion were not common findings in the chest CT scan of our patients. In terms of segmental distribution, most commonly, involvement was seen in the posterior segment of the left lower lobe. Moreover, certain segments and lobes were more frequently involved based on age and sex of patients.

The results of our study indicated that, overall, lower lobes of the lungs were more frequently involved. Specifically, the posterior segment of the left lower lobe, the superior segment of the right lower lobe and the lateral segment of the left lower lobe were predominantly involved. Consistent with our findings, a recent study by Song et al., also found lower lobe involvement in 90% of COVID-19 patients (9). Another study also pointed to the same finding that peripheral and lower lobe involvement is more common (10). Yang et al. reported the posterior basal segments of the right and left lower lobes as the most involved sites in 102 patients with COVID-19 pneumonia (11). However, they did not report the difference of segmental distribution with respect to age and sex. Our study demonstrated that certain segments and lobes are more frequently involved in females or older patients. This might provide clue to radiologists to pay particular attention to these segments when reviewing the imaging of these populations; yet, more studies are warranted for a definite conclusion. 

We observed that the majority of patients presented with peripheral distribution of lung lesions in imaging. This observation was previously reported by several other studies (10, 12, 13). However, one study has reported peripheral distribution in only 33% of investigated patients (14). Although various CT findings were observed in our cases, almost all cases had bilateral GGO in sub-pleural areas. It seems that exudative fluid accumulation within the alveolar space is the cause of this multi-lobar appearance. Since the predominant pattern is GGO, the superiority of CT scan over chest radiography is justified due to its higher resolution. It is assumed that low-dose CT scan might also be helpful; not only for diagnosis, but also for reducing radiation exposure, although further studies are needed (5). The majority of studies published so-far have also mentioned GGO, consolidation, and bronchial dilation as the main characteristic findings (4, 5, 14-19). This pattern of involvement is not pathognomonic and resembles that of other viral pneumonia, in particular, influenza. Other pathologies such as aspiration pneumonia also present with bibasilar consolidation similar to COVID-19 infection. Similar radiologic patterns are also found in interstitial lung diseases, such as organizing pneumonia or non-specific interstitial pneumonitis (NSIP), in which predominant peripheral GGO or diffuse GGO is observed. Bilateral patchy areas of GGO are also found in conditions such as lung contusion in the setting of severe chest trauma (18, 20, 21). Thus, decision making based on appropriate clinical findings is crucial for correct diagnosis.

 There was no sign of cavitation, reticulation, calcification, bronchiectasis, tree-in-bud appearance, reversed halo-sign, and nodules in any of our patients and only one patient had pleural effusion. Interestingly, the absence of these imaging features in COVID-19 has also been demonstrated by several other studies (4, 13, 14, 18). However, it is probable that these features develop in long-term, and thus, it is not possible to reach a conclusion through investigation of early imaging. Reversed halo sign is usually present in invasive fungal infections such as aspergillosis, vasculitis and even tumor metastasis; however, viral infection is also another cause of this appearance. Although this sign has been seen in some COVID-19 cases, it is considered a late finding (5, 18).

Our results revealed that less than one-fifth of patients demonstrate severe GGO on admission. It has been shown that after two weeks from initial symptoms, as consolidation begins to resolve, GGO becomes more extensive (15). This might explain the low rate of severe GGO observed in this study, as our patients were investigated within one week of symptom onset. We observed a significant association between elevated GGO score and presence of dyspnea at presentation, although it did not correlate with other symptoms such as cough and fever. Also, severity of GGO was not affected by patients’ age and sex. Notably, Zhou and colleagues also reported that dyspnea on admission was associated with increased odds of death, while fever and cough were not (22). Previous studies have stated that increase in the extent and density of GGO on CT can be used as a prognostic marker (13, 14). Several studies have displayed a significant association between higher CT score (based on extent of lung opacification) and a more severe course of COVID-19 disease (11, 23-25). In a recent study by Song et al., patients were classified into severe and non-severe groups based on clinical and imaging criteria. In this study, a self-designed CT scoring system was used as a marker for assessing the severity of the disease, showing a markedly higher CT score among patients with severe disease. Consistent with our study, they did not find a significant association between sex and disease severity; however, older cases were more likely to develop severe illness (23). Thus, although we were not able to measure the relationship between GGO severity and disease outcome, the significant association of dyspnea with a higher GGO score might indicate that the existence of this symptom at presentation indicates worse prognosis.

This study had various limitations. First, it only included adult cases. Second, it had a relatively small sample size. Moreover, we only investigated the initial CT-scans obtained on admission and findings were not controlled in terms of the exact number of days after symptom onset (15, 26). The retrospective nature of the study might have also introduced selection bias. Another major flaw was that in the majority of cases, lab tests and patient outcome were not available. In addition, evaluation of features such as cavitation and pleural effusion requires follow-up. Thus, larger multi-center cohorts along with follow-up and complete data are necessary to validate the results.

In conclusion, consistent with previous studies, we also determined that COVID-19 mainly affects the lower lobes of the lungs. Our study also confirmed that ground-glass opacities and consolidation in the lung periphery is the imaging hallmark in COVID-19 infection. Bronchiectasis, tree-in-bud appearance, discrete pulmonary nodules, cavitation, calcifications, reversed halo-sign and pleural effusions were uncommon findings. Conclusively, recognition of this pattern of chest involvement, in the proper clinical setting, is highly suggestive of COVID-19 infection. Furthermore, the association observed between dyspnea and extensive GGO on imaging might indicate that presentation with dyspnea is associated with worse prognosis compared to cough and fever. This finding could help medical staff with limited healthcare resources to predict cases with a possible worse outcome when triaging patients. Overall, imaging is valuable not only for early suspicion of the disease, but also for follow-up and evaluation of disease severity; however, further investigations are needed to shed light on the radio-clinical correlation of this disease.

**Table 1:** Baseline characteristics of studied cases

**Variables**	**Number (%)**
**Sex **	
Male	38 (60.3)
Female	25 (39.7)
**Age (year)**	
20-39	12 (19)
40-59	28 (44.4)
≥ 60	23 (36.5)
**Symptom at presentation**	
Dyspnea	33 (52.3)
Fever	45 (71.4)
Cough	30 (47.6)
Headache	6 (9.5)
Myalgia/ fatigue	9 (14.3)
Gastrointestinal symptoms	1 (1.5)

**Table 2:** Chest Computed Tomography (CT) findings of COVID-19 patients

**Location**	** Number (%)**
**Right upper lobe**	
Apical	24 (38.1)
Posterior	35 (55.5)
Anterior	23 (36.5)
**Right middle lobe**	
Lateral	41 (65.1)
Medial	11 (17.5)
**Right lower lobe**	
Superior	49 (77.8)
Posterior	47 (74.6)
Medial	40 (63.5)
Anterior	26 (41.3)
Lateral	45 (71.4)
**Left upper lobe**	
Apicoposterior	22 (34.9)
Anterior	24 (38.1)
Superior lingula	26 (41.2)
Inferior lingula	25 (39.7)
**Left lower lobe**	
Superior	39 (61.9)
Anteromedial	22 (34.9)
Lateral	48 (76.2)
Posterior	52 (82.5)
**Imaging Characteristics**	
Bilateral	53 (84.1)
Peripheral GGO	58 (92.1)
Consolidation	27 (42.9)
Peri-bronchovascular GGO	13 (20.6)
Sub pleural band	6 (9.5)
Interlobular septal thickening	4 (6.3)
Pleural effusion	1 (1.5)
**GGO severity **	
Mild	32 (50.7)
Moderate	20 (31.7)
Severe	11 (17.4)

GGO: ground-glass opacification.
